# A HLA-A2-restricted CTL epitope induces anti-tumor effects against human lung cancer in mouse xenograft model

**DOI:** 10.18632/oncotarget.6400

**Published:** 2015-11-26

**Authors:** Yuh-Pyng Sher, Su-I Lin, I-Hua Chen, Hsin-Yu Liu, Chen-Yuan Lin, I-Ping Chiang, Steve Roffler, Hsin-Wei Chen, Shih-Jen Liu

**Affiliations:** ^1^ Graduate Institute of Clinical Medical Science, China Medical University, Taichung, Taiwan; ^2^ Graduate Institute of Immunology, China Medical University, Taichung, Taiwan; ^3^ Center for Molecular Medicine, China Medical University Hospital, Taichung, Taiwan; ^4^ Division of Hematology and Oncology, China Medical University Hospital, Taichung, Taiwan; ^5^ Department of Pathology, China Medical University Hospital, Taichung, Taiwan; ^6^ National Institute of Infectious Diseases and Vaccinology, National Health Research Institutes, Zhunan, Miaoli, Taiwan; ^7^ Graduate Institute of Life Sciences, National Defense Medical Center, Taipei, Taiwan; ^8^ Institute of Biomedical Sciences, Academia Sinica, Taipei, Taiwan

**Keywords:** peptide, cytotoxic T lymphocytes, TAL6, lung cancer

## Abstract

Cancer immunotherapy is attractive for antigen-specific T cell-mediated anti-tumor therapy, especially in induction of cytotoxic T lymphocytes. In this report, we evaluated human CTL epitope-induced anti-tumor effects in human lung cancer xenograft models. The tumor associated antigen L6 (TAL6) is highly expressed in human lung cancer cell lines and tumor specimens as compared to normal lung tissues. TAL6 derived peptides strongly inhibited tumor growth, cancer metastasis and prolonged survival time in HLA-A2 transgenic mice immunized with a formulation of T-helper (Th) peptide, synthetic CpG ODN, and adjuvant Montanide ISA-51 (ISA-51). Adoptive transfer of peptide-induced CTL cells from HLA-A2 transgenic mice into human tumor xenograft SCID mice significantly inhibited tumor growth. Furthermore, combination of CTL-peptide immunotherapy and gemcitabine additively improved the therapeutic effects. This pre-clinical evaluation model provides a useful platform to develop efficient immunotherapeutic drugs to treat lung cancer and demonstrates a promising strategy with benefit of antitumor immune responses worthy of further development in clinical trials.

## INTRODUCTION

Lung cancer is one of the leading causes of malignancy-related death because of its frequency and its highly metastatic potential. Currently, immunotherapy for lung cancer is considered as a promising treatment capable of inducing systemic tumor-specific immune responses without provoking serious side effects [1, 2]. The critical factor for immunotherapy is to choose potential cancer specific antigens as targets without affecting normal tissues.

The tumor-associated antigen L6 (TAL6), is a tumor-specific antigen which is a distant member of the transmembrane-4 superfamily (TM4SF) and is often overexpressed in human lung, breast, and colon cancer tissues but not in normal tissues [3–5]. Antibody-based immunotherapy against membrane TAL6 protein was used to treat breast cancer in clinical studies [6–12], but the therapeutic effects were limited. Several strategies are used to improve immunotherapy for cancer treatment such as induction of cytotoxic T-cells (CTL) responses to lung cancer antigens [13–15]. Utilization of synthetic peptides for CTL epitopes-based vaccines are safe and easy to manufacture for clinical use. Recently, the multi-peptides vaccine (IDM-2101) was designed to induce CTLs against five tumor-associated antigens (TAAs) frequently overexpressed in NSCLC (i.e. carconoembryonic antigen (CEA), p53, Her2, and melanoma antigens (MAGE)), and it provided clinical efficacy in metastatic NSCLC [16].

Although immunotherapy or cancer vaccines to induce CTLs for NSCLC treatment has revealed promising effects [1, 2], lack of proven clinical benefits continues to block development of immunotherapy. Developing a convenient and predictive model to evaluate the CTL activity before clinical trials is critical and essential to increase the successful rate of immunotherapy. In this study, we generated a HLA-A2 transgenic mouse model and human tumor xenograft in SCID mice model to investigate the pre-clinical therapeutic effect of immunotherapy to human tumors via adoptive transfer of HLA-A2 restricted CD8^+^ CTLs recognizing human tumor antigens. Because TAL6 is a tumor specific antigen and correlates with cancer metastasis, the TAL6-derived CTL peptide was investigated to elicit tumor specific CTL responses and additive therapeutic effects with gemcitabine in pre-clinical trials.

## RESULTS

### High TAL6 protein expression in lung cancer cell lines and clinical lung tumor tissues

**Figure 1:**
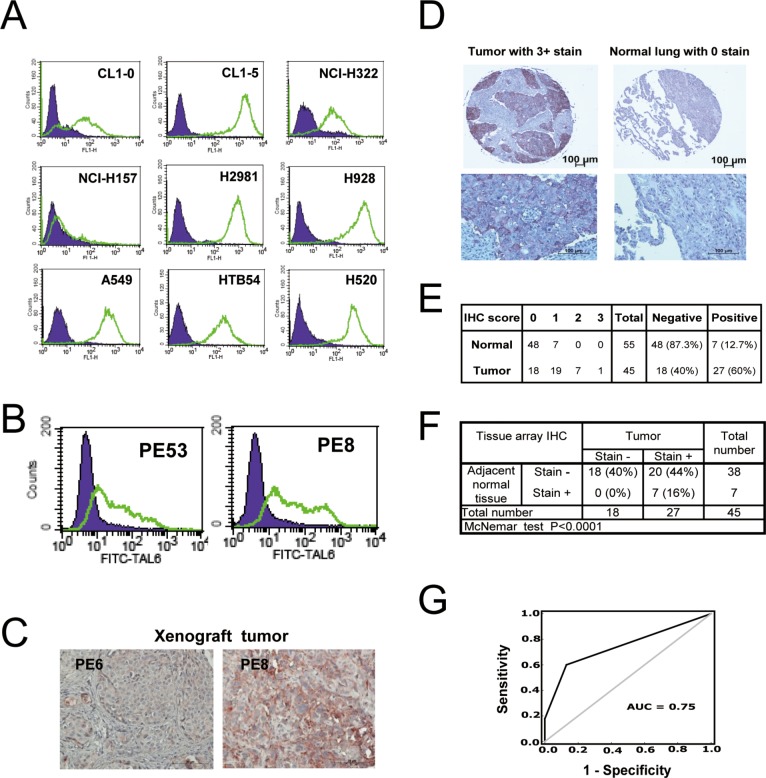
TAL6 protein is over-expressed in lung cancer cells and clinical lung tumor tissues **A.** TAL6 protein levels were measured on the cell surface by flow cytometry using mouse anti-TAL6 antibody (green line) or isotype control antibody (shaded), and then probed with goat anti-mouse FITC-conjugated antibody in 9 lung cancer cell lines. **B.** TAL6 expression in primary cultured lung cancer cells from pleural effusion was detected by flow cytometry as in panel A. PE53 and PE8 were established from two individual lung cancer patients. **C.** TAL6 expression was stained in xenograft tumors from SCID mice by subcutaneously injecting primary cultured lung cancer cells. **D.** The IHC staining from stage III lung cancer patients and cancer adjacent normal pneumonic lung tissue. An enlarged image is shown below the original IHC stains. **E.** IHC analysis of TAL6 in lung cancer tissue array scored by staining intensity from 0 to 3+ (0, negative; 1, weak; 2, moderate; 3, strong) by histologists. Positive is taken as a score of greater than 0; negative is indicated by score = 0. **F.** Analysis of IHC scores in tumor and adjacent normal tissue. **G.** ROC curve analysis for prediction of TAL6 expression in lung tumor tissues from IHC results of lung cancer tissue array. The AUC is 0.75 and the sensitivity and specificity is 70.6% and 70.6%.

To determine whether TAL6 protein was overexpressed in lung cancer cells as a tumor specific antigen for treatment, lung cancer cell lines and primary lung tumor tissues from NSCLC patients were stained with an anti-TAL6 monoclonal antibody. By using flow cytometry, high levels of TAL6 protein was detected on most of the lung cancer cell lines except NCI-H157 (Figure 1A). By comparing two cell lines from the same original cell population, we found higher TAL6 expression on CL1-5 cells with high metastatic ability as compared to parental CL1-0 cells with low metastatic ability (Figure 1A), which is consistent with previous reports that TAL6 expression on tumor cells is associated with cancer metastatic ability [21, 22]. In addition, TAL6 was detected in primary cultured lung cancer cells from lung cancer patients' pleural effusion (Figure 1B) and high TAL6 expression was maintained in human tumor xenografts in SCID mice (Figure 1C). To assess the clinical relevance of TAL6 protein expression in lung cancer patients, we performed IHC staining of TAL6 in a lung cancer tissue array, which contains 45 tumor and 55 adjacent normal tissues from Asian patients (Figure 1D). For comparison, we set a score over 0 as positive and found 60% positive staining of TAL6 in lung cancer tissues and 12.7% in normal lung tissues (Figure 1E). Notably, most normal lung tissues were in negative and the maximal score of positive staining in normal lung tissues was 1, which was only detected in paired tumors with positive staining (Figure 1F). TAL6 expression was high in lung cancer tissue but remained low in matched adjacent normal lung tissue (*P* < 0.0001; Figure 1F). Furthermore, to assess the diagnostic accuracy, we performed a receiver operating characteristic (ROC) curve analysis which is used in medicine to determine a cutoff value for the TAL6 IHC result of the tissue array [23]. Area under the curve (AUC) can range from 0.5 (random chance, or no predictive ability) to 1 (perfect discrimination/accuracy). On ROC curve analysis, the area under the curve (AUC) was 0.75 and its sensitivity and specificity was 70.6% and 70.6% (Figure 1G), indicating TAL6 expression is indeed abundant in lung cancer tissues.

### Immunization of a TAL6-derived CTL epitope can suppress tumor growth in HLA-A2 transgenic mice

**Figure 2:**
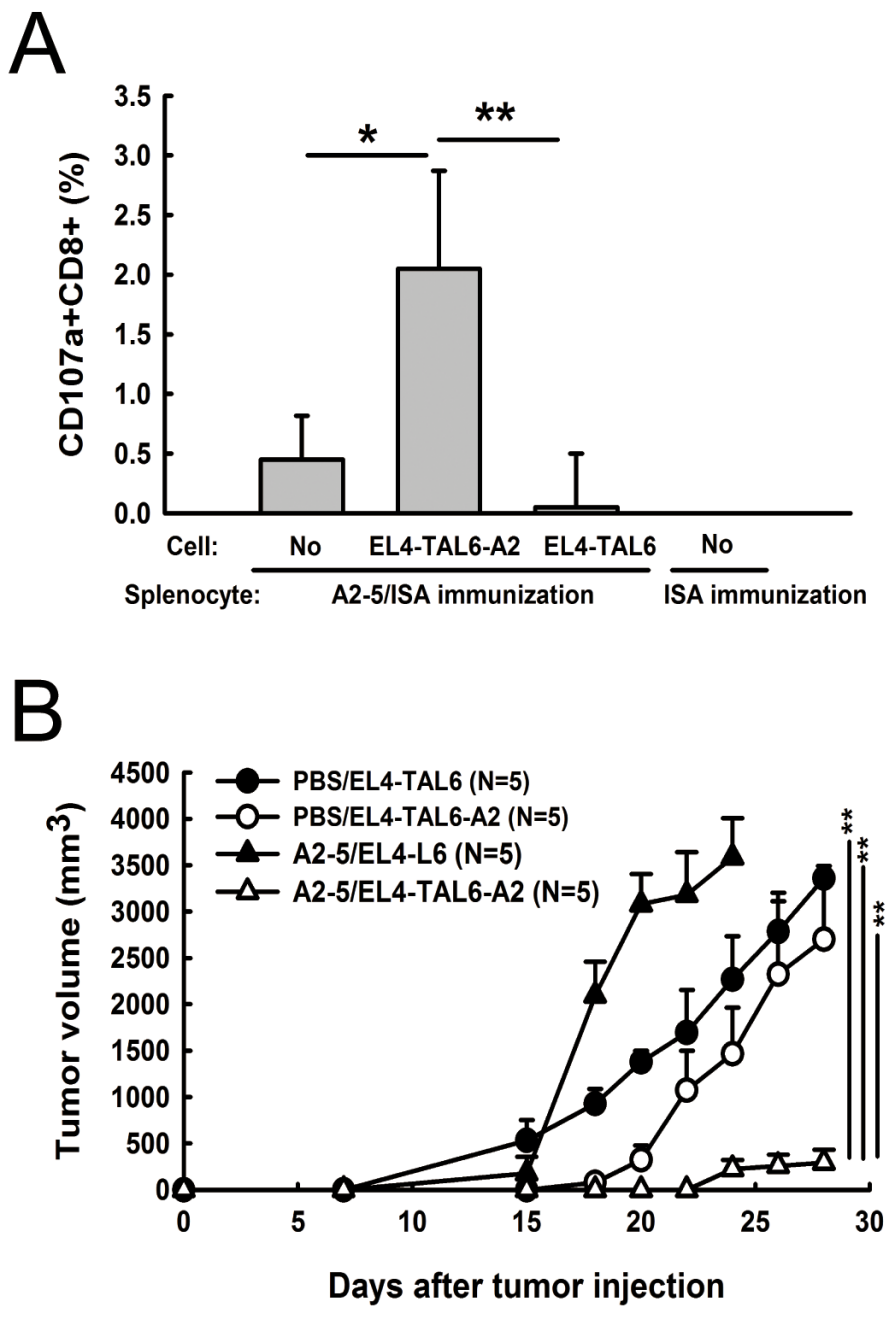
Anti-tumor effect of HLA-A2-restricted TAL6 peptide immunization in HLA-A2 transgenic mice **A.** HLA-A2 transgenic mice were immunized s.c. twice with peptide A2-5 formulated with the Th epitope peptide in ISA or ISA alone. After the final immunization, splenocytes were harvested from the mice and co-cultured with EL4-TAL6-A2 or EL4-TAL6 cells for 2 hr. The percentage of CD107a^+^CD8^+^ cells was detected by flow cytometry. Error bars, SD from three independent experiments; *, *P* < 0.05; **, *P* < 0.01. **B.** At 7 days after final immunization of PBS or peptide A2-5 in HLA-A2 transgenic mice, EL4-TAL6-A2 or EL4-TAL6 cancer cells (2 × 10^5^ cells per mouse) were injected subcutaneously and the tumor growth was monitored. Each group contains 5 mice. Error bars, SEM; **, *P* < 0.01.

In our previous study, we identified a HLA-A2 specific CTL epitope of TAL6, called peptide A2-5, that could induce HLA-A2-restricted immunity and TAL6 specific cytotoxicity of CTLs by the immunization of A2-5 formulated in incomplete Freund's adjuvant (IFA) with a universal Th epitope Pan-DR peptide against TAL6-expressing breast tumors [17]. To improve the immune-stimulatory activity of the TAL6-derived CTL epitope, we formulated peptides with adjuvant ISA (see material and method) to boost host immunity. Splenocytes from Th and A2-5 immunized A2 Tg mice were restimulated with EL4-TAL6-A2 (EL4 cells that expressed TAL6 and HLA-A2) or EL4-TAL6 (EL4 cells that expressed TAL6 alone) cells *in vitro*. We found a higher frequency of CD107a^+^CD8^+^ cells (cytolytic T cells) when cultured with EL4-TAL6-A2 cells (2.05 ± 0.40) as compared to cultures with EL4-TAL6 cells (0.5 ± 0.44). That indicated cytolytic T cells from HLA-A2 specific CTL peptides immunized mice can be activated to specifically recognize cancer cell expressed TAL6 and HLA-A2, but not to cells without HLA-A2 expression, supporting the function of HLA-A2-restricted immunity (Figure 2A). Because immunization of the A2-5 epitope could induce *ex vivo* CTL activity in the presence of HLA-A2, we further investigated the function of A2-5 immunization for *in vivo* tumor development by inoculating EL4-TAL6-A2 or EL4-TAL6 cancer cells in HLA-A2 transgenic mice. After peptide immunization, the growth of EL4-TAL6-A2 tumors was significantly suppressed compared with EL4-TAL6 tumors (Figure 2B). These results indicate that A2-5 peptide immunization can induce HLA-A2-restricted CTL responses and provide therapeutic activity in TAL6 and HLA-A2 co-expressing cancer cells.

### Improved immunization of TAL6-derived CTL epitope can suppress tumor metastases and prolong survival in HLA-A2 transgenic mice

**Figure 3:**
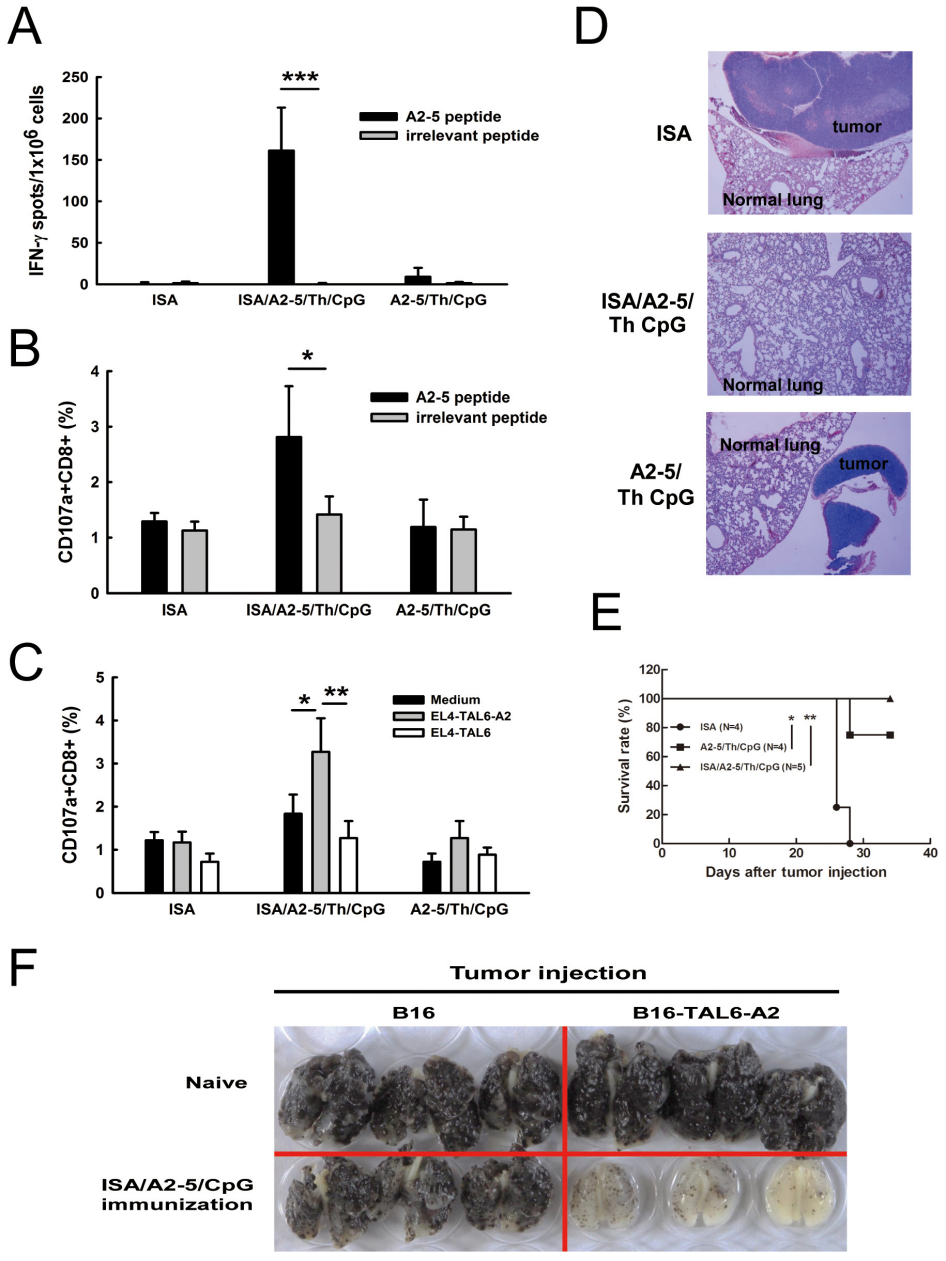
Improved immunization in HLA-A2 transgenic mice prolongs animal survival time and prevents lung metastases HLA-A2 transgenic mice were subcutaneously immunized twice with peptide A2-5 (50 μg/mouse), Th epitope peptide, CpGODN (10 μg/mouse) and ISA and then the anti-tumor effects were monitored. **A.** Splenocytes were harvested and incubated with peptide A2-5 or irrelevant peptide (10 μg/ml). IFN-γ secreting cells were detected by IFN-γ ELISPOT assay. Error bars, SD. ***P < 0.001. **B.** Splenocytes harvested from peptide immunized mice were stimulated with 10 μg/ml of peptidesfor 6 hr in the presence of PE-conjugated anti-CD107a. After stimulation, FITC-conjugated anti-CD8 antibody was used to detect CD8^+^ T cell. The percentage of CD107a^+^CD8^+^ cells in individual immunized groups was determined by flow cytometry. **C.** Irradiated EL4-TAL6-A2 or EL4-TAL6 cells (2 × 10^4^) were used to stimulate splenocytes for 2 hr. The percentage of CD107a^+^CD8^+^ cells was determined by flow cytometry as panel B described. **D.** After peptides immunization, EL4-TAL6-A2 cells (2 × 10^5^ cells) were inoculated through the i.v. routes. Lung tissues from each group were collected for tumor nodule detection 20 days after tumor implantation. **E.** Mice survival time was monitored and analyzed. *, *P* < 0.05; **, *P* < 0.01. **F.** Melanoma B16 or B16-TAL6-A2 cells (B16 cells with expression of TAL6 and HLA-A2) were intravenously injected in naïve and ISA/A2-5/Th/CpG immunized HLA-A2 transgenic mice. Whole lungs were examined.

To further improve the immunization of peptide A2-5, TLR9 ligand CpG adjuvant was included with the peptide and Montanide ISA-51 in HLA-A2 transgenic mice. Splenocytes were harvested after the final immunization and T cell activation was analyzed using the IFN-γ secreting ELISPOT assay. Formulation of ISA/A2-5/Th/CpG (combination of Montanide ISA-51, A2-5 peptide, Th peptide, and TLR9 ligand CpG) induced substantial IFN-γ secretion as compared to A2-5/Th/CpG (161.1 ± 18.38 v.s. 9 ± 3.81) (Figure 3A). Comparison of the efficacy of each adjuvant showed that ISA/A2-5/Th/CpG induced the strongest IFN-γ secretion as compared to ISA/A2-5/Th and A2-5/Th/CpG. In addition, ISA provided greater adjuvant efficacy than CpG (Supplementary Figure S1). The ISA/A2-5/Th/CpG formulation induced more CD107a^+^CD8^+^ cells, which were activated specifically with peptide A2-5 *ex vivo* (Figure 3B). Consistently, ISA/A2-5/Th/CpG induced more activated cytotoxic CD8^+^ T cells after stimulation with EL4-TAL6-A2 cells (Figure 3C). To determine whether the CTL response elicited by the peptide A2-5 can inhibit cancer metastases in HLA-A2 Tg mice, EL4-TAL6-A2 cells (2 × 10^4^) were injected intravenously to develop a tumor metastatic animal model. No metastatic tumors in lungs were observed in ISA/A2-5/Th/CpG immunized mice at 20 days after tumor inoculation, whereas lung tumor nodules were detected in the other groups (Figure 3D). Moreover, the survival was significantly prolonged in ISA/A2-5/Th/CpG immunized mice and moderately enhanced in A2-5/Th/CpG immunized mice, compared to control mice (Figure 3E). To further detect the effect of A2-5 peptide specific TAL6-derived immunity in suppressing metastasis, melanoma B16 or B16-TAL6-A2 cells (B16 cells that expressed TAL6 and HLA-A2) were intravenously injected in naïve and ISA/A2-5/Th/CpG immunized HLA-A2 transgenic mice (Figure 3F). Gross examination of whole lung specimens demonstrated that tumor metastasis to lungs were dramatically suppressed in ISA/A2-5/Th/CpG immunized mice bearing B16-TAL6-A2 tumors compared to B16 cells group. Such protection in mice was reversed in naïve mice, indicating the A2-5 immunization can induce specific TAL6-derived immunity to reduce metastasis. Thus, these results suggest that ISA/A2-5/Th/CpG could induce strong HLA-A2 specific CTL responses against cancer metastases in HLA-A2 Tg mice.

### The TAL6-derived CTL peptide immunization can suppress tumor growth in a human tumor xenograft animal model with adaptive T cell transfer from HLA-A2 transgenic mice

**Figure 4:**
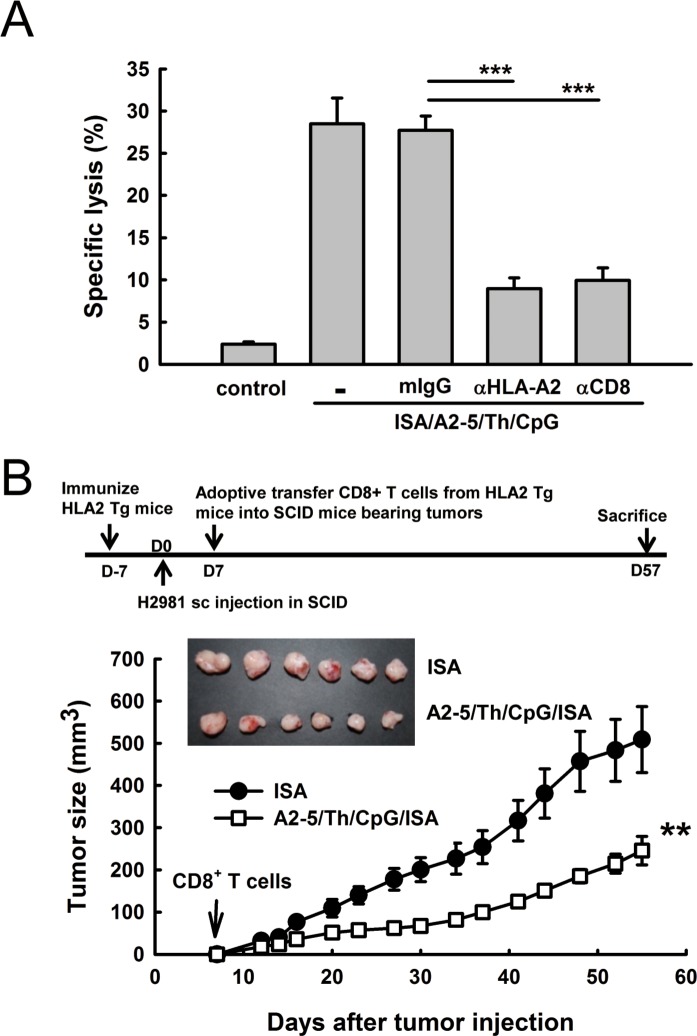
Peptide A2-5 immunization induces HLA-A2 specific cytotoxic T lymphocyteresponses in HLA-A2 transgenic mice **A.** Effector cells (splenocytes) from immunized HLA-A2 transgenic mice were stimulated with peptide A2-5 (10 μg/ml) and IL-2 (10U/ml) and subjected to a standard ^51^Cr-release assay with H2981 cells as targets. Antibodies against HLA-A2, CD8 and mIgG isotype control were incubated with effector cells at 37°C for 1 hour. CTL assays were done with 3 mice per group and observed with effector/target (E/T) ratios of 100. ***P < 0.001. **B.** The treatment schedule is shown in the top. H2981 cells (1 × 10^8^) were subcutaneously inoculated into SCID mice. Then, the purified CD8+ T cells (1 × 10^7^) from peptide A2-5 immunized HLA2 Tg mice were adoptively transferred through the i.v. route into individual SCID mouse bearing H2981 tumors on day 7. Tumor size was monitored until day 57. Each group contains 6 mice. Error bars, SEM; **, *P* < 0.01.

To determine whether the induction of HLA-A2 specific CTL responses by peptide A2-5 could inhibit the growth of human lung cancer cells, a human tumor xenograft animal model was established for assessing the therapeutic effect of the TAL6 CTL peptides elicited immune response. After identifying HLA types in human lung cancer cell lines, H2981 cells with HLA A11 and A2 were used to establish a lung cancer animal model for further treatment. After immunization of A2-5/Th/CpG/ISA twice in HLA-A2 transgenic mice, A2-5-induced CTLs destroyed lung cancer H2981 cells (28.5 ± 1.78%) and this specific cytotoxicity was inhibited with either anti-HLA-A2 (9.0 ± 0.74%) or anti-CD8 (10.0 ± 0.86%) monoclonal antibodies *in vitro* (Figure 4A). To evaluate the therapeutic efficacy of peptide-induced CTL responses in human tumor xenograft animal models, purified CD8^+^ T cells from peptide immunized HLA-A2 Tg mice were then intravenously transferred into SCID mice bearing H2981 tumors on day 7. The tumor progress in mice receiving adoptively transferred CD8^+^ T cells from A2-5/Th/CpG/ISA immunized mice was reduced significantly as compared to the control group (Figure 4B), indicating anti-tumor activity of peptide A2-5 immunized CD8^+^ CTL cells against human lung cancer cells *in vivo*.

### Combined therapy with gemcitabine and peptide A2-5 derived CTLs suppresses tumor growth and prolongs animal survival

**Figure 5:**
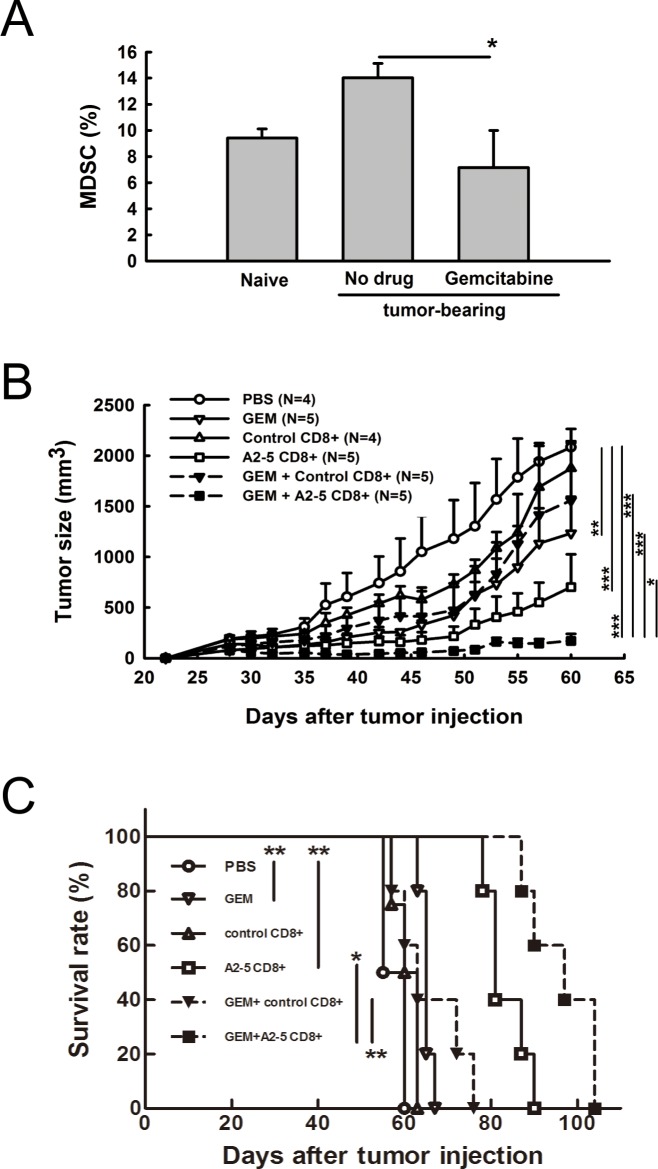
Combination of gemcitabine and peptide A2-5 induced CD8+ T cells provides anti-tumor activity and prolongs animal survival time in a human lung tumor xenograft model **A.** Lung cancer H2981 cells (1 × 10^8^) were subcutaneously injected into SCID mice. Gemcitabine was i.p. injected (3mg/mouse) into mice when tumors reach ∼ 100 mm^3^. At day 5 after gemcitabine injection, splenocytes from tumor xenografted SCID mice were isolated and the frequency of myeloid-derived suppressor cells (MDSCs) was determined by using PE-conjugated anti-GR-1 antibody and FITC-conjugated anti-CD11b antibody. Error bars, SD. *P < 0.05. **B.** Tumor size was measured at 2–3 day intervals in each group of mice. Gemcitabine was i.p. injected (3mg/mouse) on day 25 post tumor inoculation. Peptide A2-5 induced CD8^+^ T cells (1 × 10^7^) from HLA-A2 Tg mice were adoptively transferred into human tumor xenograft mice on day 30. **C.** Survival rate of mice determined with different treatments as shown in (B) Median survival time and log-rank test in SPSS analysis were analyzed.

Myeloid derived suppressor cells (MDSCs) represent one of the critical barriers to effective immune responses for detecting and eliminating cancer cells. Gemcitabine (GEM), a pyrimidine antimetabolite in clinical use for cancer treatment, has been reported to reduce the frequency of MDSCs [24]. We found that the percentage of MDSCs increased in SCID mice bearing H2981 tumors, but decreased with GEM treatment, indicating a role for GEM modulating MDSCs (Figure 5A). To determine whether combination treatment of GEM and TAL6-derived CTL peptides immunization can enhance anti-cancer activity, CTLs derived from peptide-immunized HLA-A2 transgenic mice were adoptively transferred into SCID mice bearing H2981 tumors with or without GEM treatment. Although treatment with GEM or adoptive transfer of peptide A2-5 vaccinated CD8^+^ T cells provided significant anti-tumor effects, combination of these two treatments reduced the tumor size more dramatically and more effectively prolonged mice survival (Figure 5B and 5C). Comparison of the median survival time showed that the GEM+A2-5 CD8^+^T cells group had the longest survival of 97 days, better than A2-5 CD8^+^ (81 days), GEM+control CD8^+^ (63 days), GEM (65 days), control CD8^+^ (60 days), and PBS (55 days) groups.

**Figure 6:**
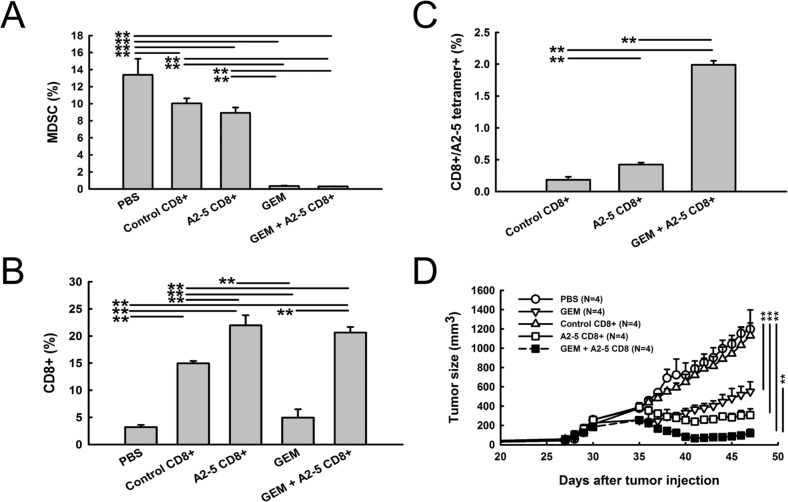
Reduced MDSC and increased peptide A2-5 induced CD8+ T cells were detected in xenograft tumors After treatment as described in Figure 5, H2981 tumors were collected on day 26 from SCID mice bearing human lung tumor xenografts. All tumors were subjected to enzymatic digestion to obtain single-cell suspensions for cell population analysis. **A.** The percentage of MDSC was detected with FITC-conjugated anti-GR-1 antibody and PE-conjugated anti-CD11b antibody. **B.** Tumor infiltrated CD8^+^T cells were stained with FITC-conjugated anti-CD8. **C.** The percentage of A2-5-HLA-A2 tetramer-binding cells was detected by flow cytometry. Error bars show mean and SD. **P < 0.01. **D.** H2981 cancer cells (1 × 10^8^) were subcutaneously injected in SCID mice. After 30 days, gemcitabine was i.p. injected (3mg/mouse). Isolated CD8^+^ T cells (1 × 10^7^) were adoptively transferred into SCID mice bearing xenograft tumors at day 35.

To investigate further whether MDSC elimination and peptide A2-5 derived CTLs are critical for the additive effect of combined antitumor therapy, the tumor-infiltrated cell population was analyzed. The percentage of MDSC was significantly decreased in GEM and GEM combined with the peptide A2-5 vaccinated CD8^+^ T cells groups (Figure 6A). Large numbers of tumor-infiltrated CD8^+^T cells were detected in mice receiving control CD8+T cells, A2-5 vaccinated CD8^+^ T cells, and GEM combined with a peptide A2-5 vaccinated CD8^+^ T cells groups (Figure 6B). However, the percentage of A2-5-HLA-A2 tetramer-binding cells, which indicate the specific A2-5 derived CTLs, was greatly increased in the mice treated with both GEM and peptide A2-5 vaccinated CD8^+^ T cells as compared to the A2-5 CD8^+^ group (Figure 6C), even though the two groups had similar CD8^+^ T cells percentages (Figure 6B). It is likely that reduction of MDSC by gemcitabine preserved the specific A2-5 derived CD8^+^ T cells in tumors. These results suggest improved therapeutic application with GEM and adoptive transfer of peptide A2-5 vaccinated CD8^+^ T cells. Furthermore, to evaluate if the vaccine could suppress established tumors, we treated tumors after 30 days of cancer cell inoculation. GEM or adoptive transfer of peptide A2-5 vaccinated CD8^+^ T cells suppressed tumor growth, and combination treatment showed additive anti-tumor activity (Figure 6D).

## DISCUSSION

Although TAL6 was considered as a target for antibody-based immunotherapy against breast and lung cancer during the past decade, the therapeutic effects of TAL6 antibody-based immunotherapy are limited in humans. Alternatively, T cell-based immunotherapy may be a feasible approach for cancer treatment. Recently, we have identified a HLA-A2-restricted CTL epitope of TAL6 that can stimulate CTL activation to kill TAL6-expressing MCF-7 breast cancer cells *in vitro* [17]. In this study, we further extended this T cell-based immunotherapy for lung cancer treatment. TAL6 protein was previously detected with antibody in most NSCLC tissues [4], but the expression frequency of TAL6 protein in lung cancer is less reported in Asian patients. In this report, we found that TAL6 was highly expressed in over 80% of lung cancer tissues in Asian patients, suggesting thatTAL6 could be a tumor antigen. The CTL epitope A2-5 peptide induced anti-tumor effects against lung cancer in HLA-A2 transgenic mice, and adoptive transfer of CD8+ T cells from peptide A2-5 immunized mice into SCID mice inhibited tumor growth of human lung cancer xenografts. More importantly, additive improvement of the therapeutic effects with T cell-based immunotherapy and gemcitabine chemotherapy was observed in a human cancer xenograft model. These results demonstrate that peptide A2-5 immunization to elicit CTL responses is feasible for lung cancer treatment.

Immunotherapy to induce cytotoxic T lymphocyte (CTL) activity is important to inhibit human tumor growth and is a promised strategy. The deficiency of good evaluation systems for pre-clinical therapeutic studies is a major challenge for T cell-based immunotherapy assessment due to the human HLA restriction of therapeutic CTL peptides. Here, we developed a convenient model to evaluate CTL activity before clinical trials in HLA-A2 transgenic mice immunized with different formulations. ISA-51 adjuvant has been used in human clinical trials and is safer than incomplete adjuvant (IFA). We found that formulation with ISA-51, peptide A2-5, a Th epitope and TLR 9 agonist (CpG) (ISA/A2-5/Th/CpG) provided stronger anti-tumor effects than formulation with ISA/A2-5/Th. The results demonstrated that emulsion type adjuvant ISA may prolong the CpG release and enhance CTL responses. The findings are similar to our previous reports that emulsion type adjuvant PELC and PELA73 could enhance CTL responses and anti-tumor effects in the presence of CpG ODN [25, 26]. Thus, combination of emulsion type adjuvant and a TLR9 agonist is a potent formulation for peptide-based therapeutic vaccines for cancer therapy.

We proved that adoptive transfer of A2-5-induced CTL from HLA-A2 Tg mice could inhibit H2981 cells growth in SCID mice, supporting the notion that peptide A2-5 could be effective in humans. However, there is still room to improve the anti-tumor effects of peptide A2-5 immunization. The tumor-infiltrating immunosuppressive cells impair CTL functions in the tumor microenvironment and are major barriers for cancer therapy. To overcome the immunosuppressive microenvironment, several approaches are aimed at depleting immunosuppressive cells including antibody deletion [27], chemotherapeutic drugs [28, 29], or TLR ligand modulation [20, 30, 31]. We selected gemcitabine for combination therapy because gemcitabine has been reported to inhibit Tregs and MDSCs in the tumor microenvironment for enhancement of anti-tumor immunity [32–34]. We confirmed that the number of MDSCs was reduced in human tumor-bearing SCID mice treated with gemcitabine. Gemcitabine combined with CD8 T cells adoptively transferred from A2-5 immunized HLA-A2 Tg mice significantly suppressed the growth of human tumors in SCID mice. These observations implied that combination of chemo-drugs and CD8^+^ T cell-based immunotherapy might benefit cancer patients.

We provide direct evidence that A2-5-induced CD8^+^ T cells are able to kill human lung cancer that expressed high-levels of TLA6 *in vitro* and *in vivo*. In addition, we showed that peptide formulated with ISA/Th/CpG elicited strong anti-tumor immunity. To achieve the maximum anti-tumor ability, selection of suitable adjuvants is important. In the future, the Th epitope could be conjugated with the CTL epitope to simplify the peptide-synthetic process. Furthermore, multiple CTL epitopes from different functional tumor-associated antigens could be added to boost the CTL activity.

Recently, TAL6 was found to be critical for endothelial cell function and tumor angiogenesis [35] and could be a vascular therapeutic target in cancer therapy [36]. Another TAL6 family protein, TM4SF5, is overexpressed in hepatocellular carcinoma and colon cancer [37, 38]. The TM4SF5-specific monoclonal antibody could inhibit colon cancer growth in a mouse model [39]. These studies demonstrated that TAL6 family proteins may be good targets for antibody-based cancer immunotherapy. Therefore, our current study provides a promising strategy to facilitate successful cancer therapy for cancer that expresses TAL6 family proteins.

## MATERIALS AND METHODS

### Detection of TAL6 expression

TAL6 protein on cells was detected by flow cytometry using a mouse monoclonal antibody against TAL6 [17]. A rabbit anti-human TM4SF1 antibody (Sigma) was used to detect TAL6 in lung cancer tissues by immunohistochemical (IHC) staining with horseradish peroxidase-conjugated avidin biotin complex (ABC) from the Vectastain Elite ABC Kit (Vector Laboratories, Burlingame, CA) and AEC chromogen (Vector Laboratories). The sections were counterstained with hematoxylin and mounted. An Asian lung cancer tissue array was purchased from US Biomax (LC1006, Rockville, MD). All stainings were evaluated by experienced histologists.

### Animals and Cell lines

Human lung adenocarcinoma cell lines CL1-0 (low invasiveness) and CL1-5 (high invasiveness) were established from the same lung cancer origin [18]. Primary cultured cell lines, PE53 and PE8, were established from the pleural effusion of two individual adenocarcinoma lung cancer patients with written informed consent from each patient. Other lung cancer cell lines were obtained from the Bioresource Collection and Research Center (Hsin-Chu, Taiwan). The EL4-TAL6-A2 cells stably express TAL6 and HLA-A2 in EL4 cells. The EL4-TAL6 cells are EL4 cells that stably express TAL6. These cells were cultured in RPMI-1640 medium supplemented with 10% fetal bovine serum (FBS). HLA-A2 transgenic mice were kindly provided by professor Show-Li Chen (National Taiwan University, Taiwan). All animal experiments were performed in specified pathogen-free (SPF) conditions under protocols approved by the Animal Committee of the National Health Research Institutes (NHRI).

### Peptide immunization of HLA-A2 transgenic mice

Peptide A2-5 (LLMLLPAFV) from the TAL6 protein was identified as an HLA-A2 specific CTL epitope in our previous study [17]. An irrelevant peptide (LYLTQDLFL, from the spike protein of SARS CoV) was included for immunization. Peptides with purity > 90% were synthesized by the peptide synthesis core facility of the National Health Research Institutes (NHRI) in Taiwan. All peptides were dissolved in 100% DMSO at 10 mg/mL as stock solutions, stored at −80°C, and analyzed by HPLC and mass spectrometry to verify their purity. Peptide immunization was performed as previously described [17]. In brief, 1 mg CTL peptide and 1 mg Th epitope peptide (PADRE: AKFVAAWTLKAAA) in 0.5 ml PBS were mixed with 0.5 ml of Montanide ISA 51 (ISA, from SEPPIC company) and then the mixtures were injected s.c. in HLA-A2 Tg mice twice at a 7-day interval. The splenocytes were collected 7 days after the second injection for further ELISPOT assay or CD107a^+^ CD8^+^ cells detection.

### ELISPOT assay

The ELISPOT assay was performed as previously described [19]. In brief, 5 × 10^5^ spleen cells with 10 μg/ml of the indicated peptides were added to a 96-well PVDF-membrane plate coated with anti-IFN-*γ* antibody. Spots were developed using a 3-amine-9-ethyl carbazole (AEC, Sigma) solution. The reaction was stopped after 4-6 minutes by running the plate under tap water. The spots were then counted using an ELISPOT reader (Cellular Technology Ltd., Shaker Heights, OH).

### Tumor model and treatment

HLA-A2 Tg mice were injected s.c. twice at a 2-week interval with A2-5 formulated in ISA-51 with 50 μg of Th and 10 μg of CpG. CpG was purchased from GeneDirex. It consisted of a sequence of 5′-TCCATGACGTTCCTGACGTT-3′ with a phosphorothioate backbone. The CD8^+^ T cells were harvested by mouse CD8^+^ T cell Dynabeads (invitrogen). The EL4-TAL6 or EL4-TAL6-HLA-A2 cells (2 × 10^5^) were inoculated s.c. at the opposite site of peptide injection seven days after the second immunization. Tumor size was measured 3 times per week by using the formula: tumor volume = length × width × width/2. In a metastatic mouse model of melanoma, HLA-A2 Tg mice were immunized with ISA/A2-5/Th/CpG by the above procedure and then B16 or B16-TAL6-A2 (5 × 10^5^) cells were intravenously injected at 7 days post final immunization. Lung tissues were collected and fixed in 8% formalin for detecting the tumor nodules after 20 days of tumor inoculation. In human tumor xenograft model, human lung cancer H2981 cells (1 × 10^8^) were subcutaneously injected in SCID mice (BioLASCO, Taiwan) to generate a human tumor xenograft animal model. Purified CD8+ T cells (1 × 10^7^) from peptide-immunized HLA2 Tg mice were intravenously delivered into SCID mice on day 7 post tumor inoculation. In combination therapy, 22 or 30 days after H2981 inoculation, gemcitabine was administered i.p. as a single dose (3mg/mouse) in the cancer xenotransplanted mice. At 5 days after gemcitabine administration, isolated CD8^+^ T cells (1 × 10^7^) were adoptively transferred into mice.

### CD107a cytotoxicity assay

The CD107a cytotoxicity assay has been described in previous reports [17]. HLA-A2 Tg mice were injected s.c. twice with the indicated peptide (50 μg/ml) emulsified in ISA in the presence of Th peptide (50 μg/ml) and CpGODN (10 μg/mouse). On day 7 after the second immunization, splenocytes were harvested and then suspended at 2 × 10^7^ cell/ml in medium that contained 10 μg/ml of the indicated peptides (50 μg/ml) or cells (2 × 10^6^ cell/ml) and PE-conjugated anti-CD107a monoclonal antibody (1:100) in 96-well round-bottom plates. After 2 hours of incubation at 37°C, brefeldin A (10 μg/ml) and monensin (0.66 μg/ml) were added for another 2-6 hours. The plates were washed with PBS containing 0.1% FBS, and rat anti-mouse Fc antibody (1:100) was added for 5 minutes, followed by addition of the FITC-conjugated rat anti-mouse CD8 antibody (1:100) for 30 minutes. The cytotoxic CD107a^+^ CD8^+^ cells were analyzed on a flowcytometer (FACS Calibur, BD Bioscience).

### ^51^Cr release cytotoxicity assays

To analyze T cell cytotoxicity, HLA-A2 transgenic mice were subcutaneously immunized twice with peptide (50 μg/mouse), CpGODN (10 μg/mouse) and ISA. 7 days after the final immunization, spleens and lymph nodes were isolated and stimulated with peptide A2-5 (10 μg/ml) and IL-2 (10U/ml) for 5 days as effector cells. Effector cells were incubated with mouse anti-HLA-A2, rat anti-CD8 and mouse or rat IgG isotype control antibodies at 37°C for 1 hour. H2981 cells (5 × 10^6^) were labeled with chromium (100 μCi) for 2 hour as target cells. The target cells (5 × 10^3^/well) were incubated at E: T cell ratios of 100:1 in a final volume of 100 μl/well at 37°C for 5 hours. Supernatants (100 μl) were harvested, and ^51^Cr release was measured. Spontaneous release was measured in wells containing target cells alone. Maximum ^51^Cr release in target cells was measured by adding 2% Triton X-100. Specific lysis (%) was calculated as: 100x (test ^51^Cr release - spontaneous ^51^Cr)/(maximum ^51^Cr release - spontaneous ^51^Cr).

### Tumor-infiltrating cells analysis

Cell surface marker staining and flow cytometry were performed as previously described [20]. Briefly, cell suspensions from tumor-bearing mice were mechanically disrupted into fragments at 24 hr after CD8^+^ T cells adoptively transferred. Cells were stained with PE-conjugated anti-CD11b and PE-Cy7-conjugated anti-Gr-1 antibodies to quantify MDSCs, FITC-conjugated CD8 to quantify CD8 T cells, and a PE-conjugated HLA-A2/A2-5 tetramer to detect A2-5-specific T cells. The cell populations were determined via flow cytometry (FACSCalibur, BD Bioscience, San Jose, CA, USA). All data were acquired using a FACSCalibur device and were plotted using FCS express version 3.0 software (research edition, De Novo software™).

### Statistical analysis

The statistical significance of differences between mean values of the experimental groups was determined using one way analysis of variance (ANOVA). The differences were considered statistically significant if the *P* value was < 0.05.

## SUPPLEMENTARY FIGURE


