# Supplementary material for: A HLA-A2-restricted CTL epitope induces anti-tumor effects against human lung cancer in mouse xenograft model
Source: Oncotarget. 2015 Nov 26;7(1):671–83. doi: 10.18632/oncotarget.6400 (PMC4808025; doi:10.18632/oncotarget.6400)
Supplement: Supplementary file 1 [file oncotarget-07-0671-s001.pdf]

SUPPLEMENTARY FIGURE

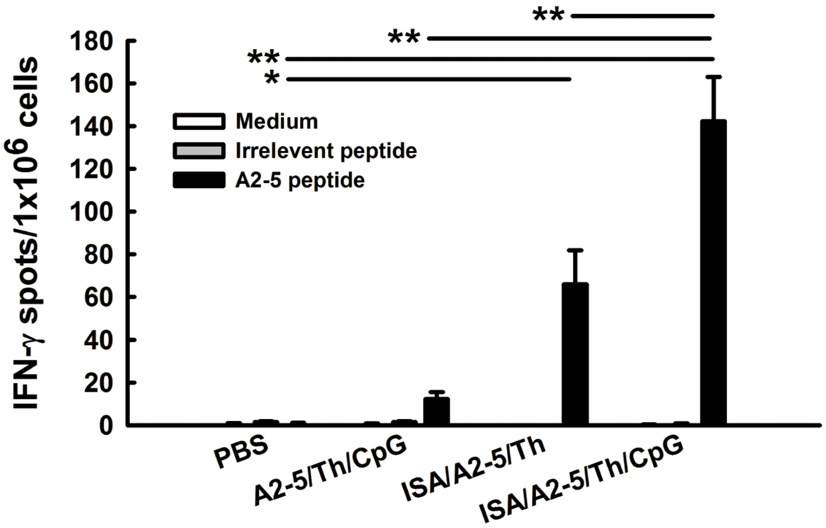

Supplementary Figure S1: Immunization of ISA/A2–5/Th/CpG elicited high IFN-γ secretion cells in HLA-A2 transgenic mice. HLA-A2 transgenic mice were subcutaneously immunized twice with A2–5 (50 µg/mouse) and CpGODN (10 µg/mouse)/ISA. Splenocytes were harvested and incubated with various peptides (10 µg/ml). Error bars, SD. $*P < 0.05$. $**P < 0.01$.
